# Alpinetin attenuates inflammatory responses by suppressing TLR4 and NLRP3 signaling pathways in DSS-induced acute colitis

**DOI:** 10.1038/srep28370

**Published:** 2016-06-20

**Authors:** Xuexiu He, Zhengkai Wei, Jingjing Wang, Jinhua Kou, Weijian Liu, Yunhe Fu, Zhengtao Yang

**Affiliations:** 1 College of Veterinary Medicine, Jilin University, Jilin, Changchun 130062, People’s Republic of China

## Abstract

Alpinetin, a composition of *Alpinia katsumadai Hayata*, has been reported to have a number of biological properties, such as antibacterial, antitumor and other important therapeutic activities. However, the effect of alpinetin on inflammatory bowel disease (IBD) has not yet been reported. The purpose of this study was to investigate the anti-inflammatory effect and mechanism of alpinetin on dextran sulfate sodium (DSS)-induced colitis in mice. *In vivo*, DSS-induced mice colitis model was established by giving mice drinking water containing 5% (w/v) DSS for 7 days. Alpinetin (25, 50 and 100 mg/kg) were administered once a day by intraperitoneal injection 3 days before DSS treatment. *In vitro*, phorbol myristate acetate (PMA)-differentiated monocytic THP-1 macrophages were treated with alpinetin and stimulated by lipopolysaccharide (LPS). The results showed that alpinetin significantly attenuated diarrhea, colonic shortening, histological injury, myeloperoxidase (MPO) activity and the expressions of tumor necrosis factor (TNF-α) and interleukin (IL-1β) production in mice. *In vitro*, alpinetin markedly inhibited LPS-induced TNF-α and IL-1β production, as well as Toll-like receptor 4 (TLR4) mediated nuclear transcription factor-kappaB (NF-κB) and NOD-like receptor protein 3 (NLRP3) inflammasome activation. In conclusion, this study demonstrated that alpinetin had protective effects on DSS-induced colitis and may be a promising therapeutic reagent for colitis treatment.

Inflammatory bowel disease (IBD) is a noninfectious, chronic and relapsing inflammation of the gastrointestinal characterized by recurrent and long-lasting episodes of diarrhea and abdominal pain, which primarily manifests as ulcerative colitis (UC) and Crohn’s disease (CD)^1^. The process of these two diseases consisted of a number of reactions, such as enetic, environmental, and immunological factors. In addition, it occurs more commonly in Western countries with causing the greatest economic loss, meanwhile reducing in the quality of life and increasing the risk of developing colorectal cancer^2^,^3^. Currently, the treatment for IBD mainly depends on anti-inflammatory drugs, immune system suppressors and antibiotics, which existed many resistances problem included major adverse events and poor treatment responses. Therefore, the development of novel therapies for IBD is imminently needed.

Alpinetin (7-hydroxy-5methoxy- 2-(4-hydroxyphenyl) -4H-1-benzopyran-4-one, C_16_H_14_O_4_, 270.27996, Fig. 1), a novel plant flavonoid isolated from Alpinia katsumadai Hayata, is a traditional Chinese medicine. It has been reported that alpinetin has antibacterial, anti-tumor and other important therapeutic activities^4^,^5^. In previous studies, the protective effects of alpinetin on mastitis in mice and acute lung injury in mice have been confirmed^6^,^7^. However, there is little information about the effect of alpinetin on DSS-induced colitis in mice. This research aims to investigate the anti-inflammatory effect and mechanism of alpinetin in DSS-induced colitis in mice.

## Results

### *In vivo* study.

#### Alpinetin attenuated DSS-induced colitis

**Figure 2:**
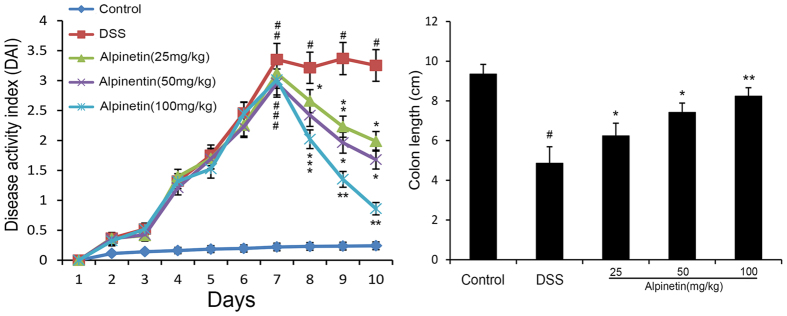
Alpinetin ameliorated the progression of DSS-induced colitis in mice. (**A**) Mice were administered 5% DSS in drinking water (ad libitum) for 7 days with/without alpinetin (25, 50 and 100 mg/kg/day p.o.). Changes in DAI were evaluated daily. (**B**) Colons were obtained after 7 days of DSS administration and their lengths were measured. The values presented are the mean ± S.E.M (n = 10 in each group). Number sign (#) indicates P < 0.01 vs. control group. Single asterisk (*) indicates P < 0.05, anddouble asterisks (**) indicate P < 0.01 vs. DSS group.

To determine the therapeutic potential of alpinetin, we established the mouse model of DSS-induced colitis, characterized by significant appearance of weight loss, diarrhea or loose feces, and visible fecal blood. The protective effect of alpinetin on DSS-induced colitis was assessed using a disease activity index (DAI). Our results showed that DAI scores were significantly increased in the DSS group compared with the control group. However, alpinetin (25, 50 and 100 mg/kg) decreased the DAI score in a dose-dependent manner as compared with the DSS. Moreover, we also observed that alpinetin (25, 50 and 100 mg/kg) dose-dependently prevented the shortening of the colon which the length of the colon is a marker negatively relevant to the severity of colitis (Fig. 2).

#### Effect of alpinetin on histopathological changes

**Figure 3:**
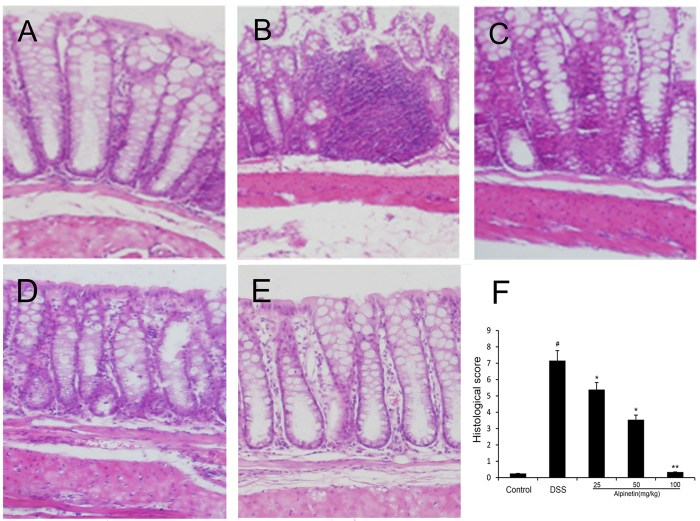
Effect of alpinetin on histopathologic changes in colon tissues in DSS-induced mice colitis. Colon tissue of control group (**A**), the DSS group (**B**), the DSS + alpinetin 25 mg/kg group (**C**), the DSS + alpinetin 50 mg/kg group (**D**), the DSS + alpinetin 100 mg/kg group (**E**) and the histological scores (**F**).

In this study, the severity of colitis was further evaluated by histopathological analysis using H.E staining. Compared with the control group (Fig. 3A), the DSS group (Fig. 3B) exhibited severe damage in the surface epithelium, a pronounced decrease in the number of crypts, and infiltration of inflammatory cells, overcoat in middle and rectum sections. However, pretreatment with alpinetin dose-dependently attenuated pathological changes by DSS-induced (Fig. 3C–E). The histopathological injury scores were significantly decreased (Fig. 3F).

#### Effect of alpinetin on MPO activity

**Figure 4:**
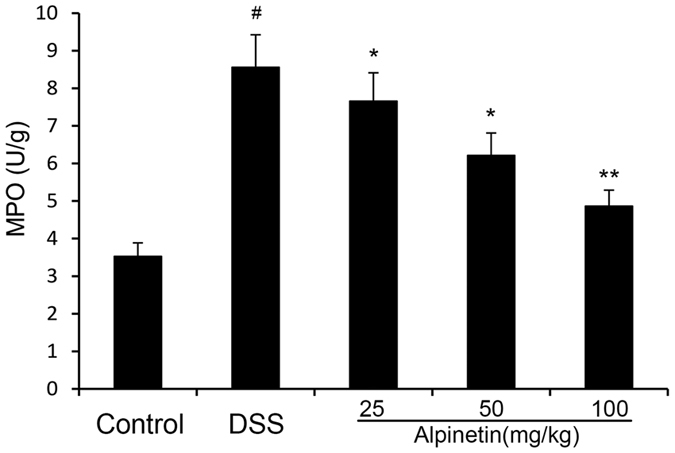
Effects of alpinetin on MPO activition in colon tissues of DSS-induced colitis. The values presented are the mean ± S.E.M (n = 10 in each group). Number sign (#) indicates P < 0.01 vs. control group. Single asterisk (*) indicates P < 0.05, anddouble asterisks (**) indicate P < 0.01 vs. DSS group.

MPO, a marker of inflammation, tissue injury and neutrophil infiltration, reflects the level of inflammation and oxidative stress. After treatment with DSS, the MPO activity was significantly increased in the DSS group, and this was dose-dependently reduced through pretreatment with alpinetin at the doses of 25, 50 and 100 mg/kg (Fig. 4).

#### Effect of alpinetin on the level of inflammatory cytokines

**Figure 5:**
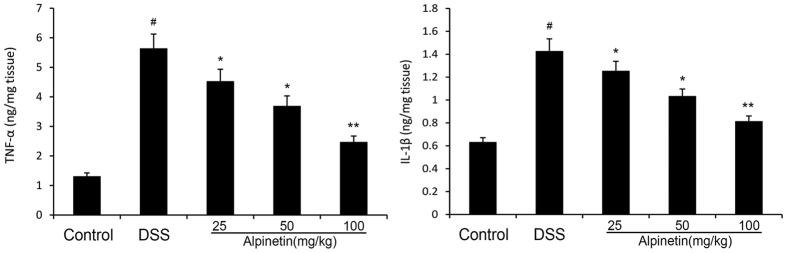
Effects of alpinetin on the levels of TNF-αand IL-1β in the homogenate of DSS-induced mice colon tissues. The values presented are the mean ± S.E.M (n = 10 in each group). Number sign (#) indicates P < 0.01 vs. control group. Single asterisk (*) indicates P < 0.05, anddouble asterisks (**) indicate P < 0.01 vs. DSS group.

The levels of pro-inflammatory cytokines TNF-α and IL-1β were measured by ELISA. Our results demonstrated that the levels of pro-inflammatory cytokines TNF-α and IL-1β were significantly increased after DSS administration, whereas administration of alpinetin significantly reduced the levels of these pro-inflammatory cytokines in a dose-dependent manner (Fig. 5).

### *In vitro* study

#### Effect of alpinetin on cell viability

**Figure 6:**
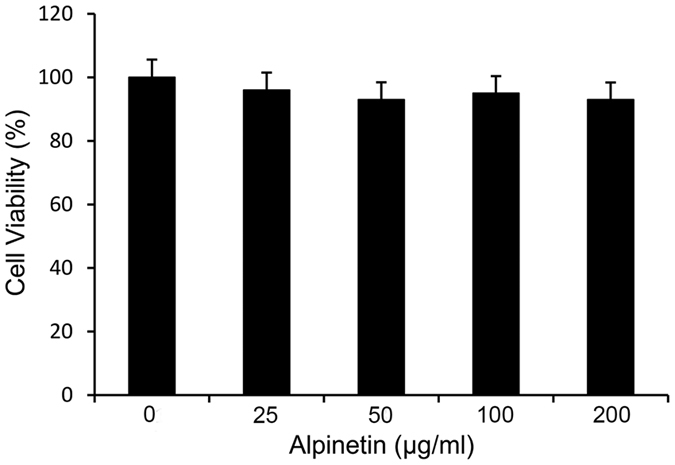
Effects of alpinetin on THP-1 cells viability. THP-1 cells were exposed with or without alpinetin (50, 100 and 200 μg/ml) for 24 h. The cells were not affected by the alpinetin treatment.

The potential cytotoxicity of alpinetin was assessed by the MTT assay. The results showed that cell viability was not affected by the alpinetin (50, 100 and 200μg/ml) treatment (Fig. 6).

#### Effect of alpinetin on cytokines

**Figure 7:**
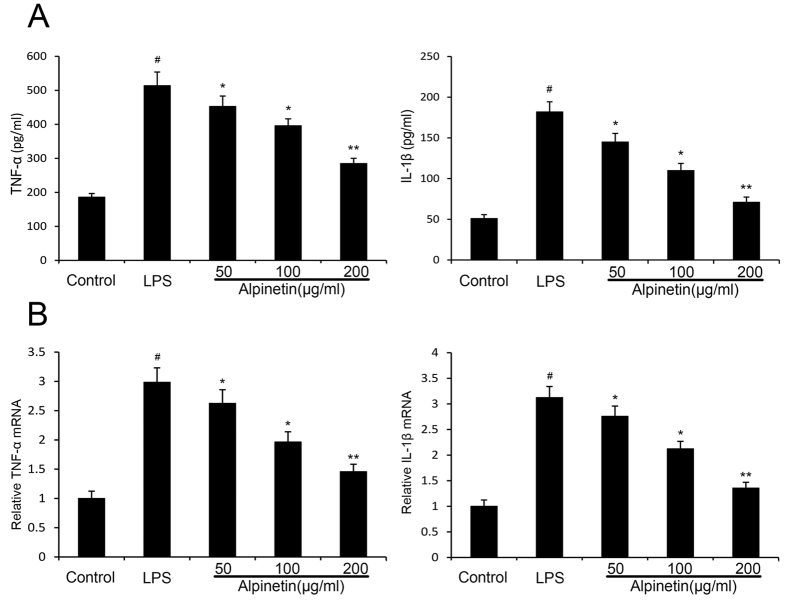
Effects of alpinetin on the levels of TNF-α and IL-1β in the cell supernatants. The productions of TNF-α and IL-1β were detected by ELISA (**A**). The mRNA levels of TNF-α and IL-1βwere detected by RT-PCR (**B**).The values presented are the mean ± S.E.M (n = 10 in each group). Number sign (#) indicates P < 0.01 vs. control group. Single asterisk (*) indicates P < 0.05, anddouble asterisks (**) indicate P < 0.01 vs. LPS group.

To further explore the anti-inflammatory mechanism of alpinetin, we investigated the productions of TNF-α and IL-1β by ELISA and the mRNA levels of TNF-α and IL-1β by RT-PCR. Our results showed that the productions and mRNA levels of pro-inflammatory cytokines TNF-α and IL-1β increased significantly in LPS treatment. However, the productions and mRNA levels of TNF-α and IL-1β decreased significantly in a dose-dependent manner when the cells were treated with alpinetin (50, 100 and 200 μg/ml) (Fig. 7).

#### Effect of alpinetin on NF-κB signaling pathway

**Figure 8:**
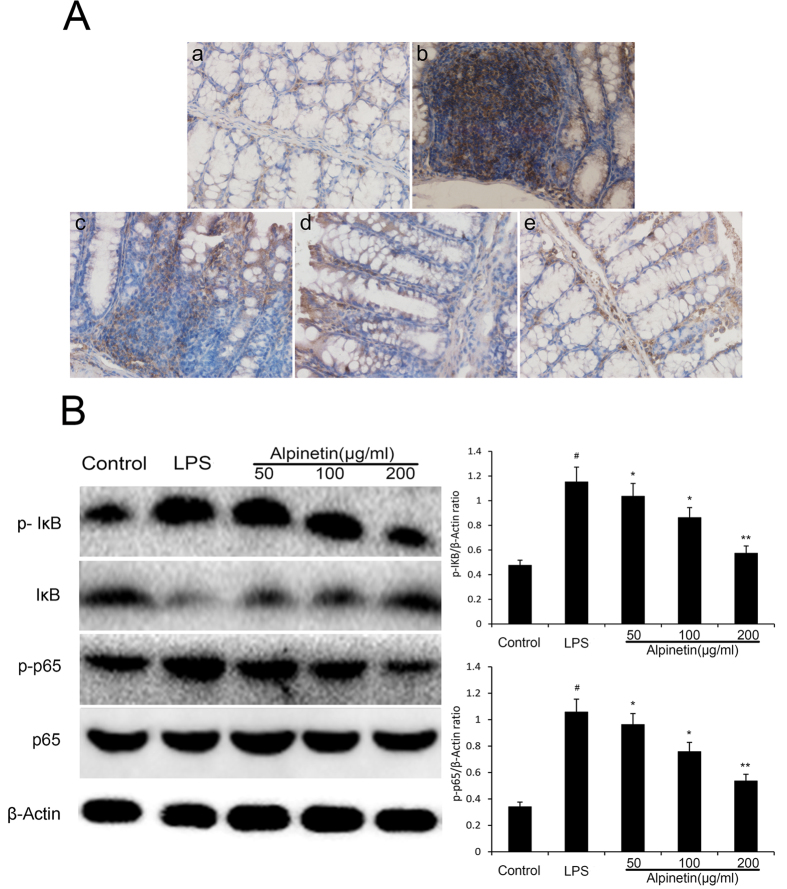
Alpinetin inhibited NF-κB pathway. Colon tissue was collected to determine the NF-κB positive signal by immunohistochemistry (**A**). Protein levels of NF-kB p65 and IκBα in THP-1 cells were determined by Western blotting (**B**). The values presented are the mean ± S.E.M (n = 10 in each group). Number sign (#) indicates P < 0.01 vs. control group. Single asterisk (*) indicates P < 0.05, anddouble asterisks (**) indicate P < 0.01 vs. LPS group.

To investigate the possible molecular mechanism of alpinetin, the activation of NF-κB signal pathways were evaluated. NF-κB localization in the colon was illustrated in Fig. 8A. NF-κB positive signals were observed to be obviously increased in the colon after DSS administration compared with the control group. Notably, NF-κB positive signals in the colon were significantly reduced by pretreatment with alpinetin. In THP-1 cells, application of LPS led to phosphorylation of p65 and IκBα phosphorylation in the NF-κB signaling pathway. However, pretreatment with alpinetin significantly inhibited their phosphorylation by LPS (Fig. 8B).

#### Effect of alpinetin on the activity of TLR4

**Figure 9:**
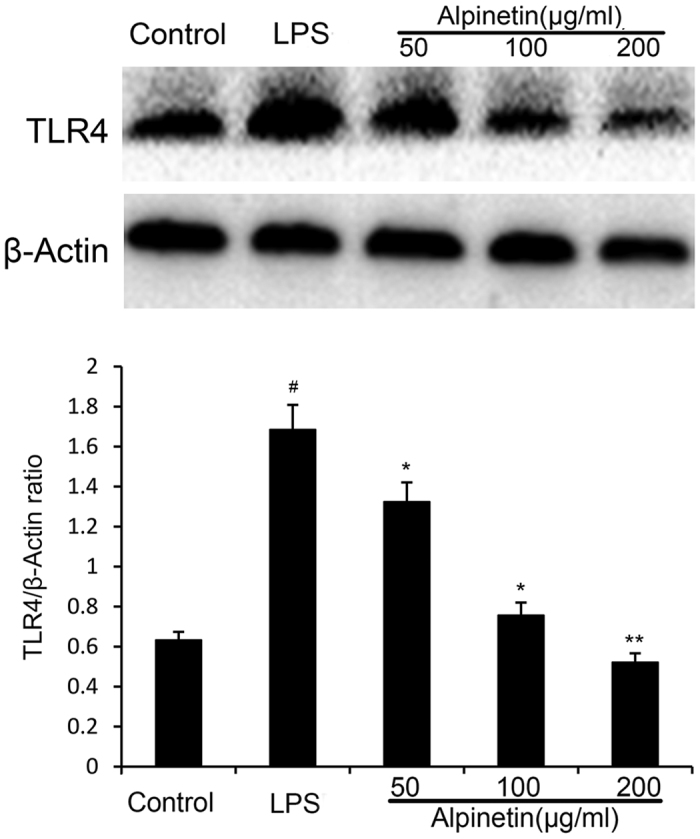
Alpinetin inhibited LPS-induced activation of TLR4 expression with Western blotting. The values presented are the mean ± S.E.M (n = 10 in each group). Number sign (#) indicates P < 0.01 vs. control group. Single asterisk (*) indicates P < 0.05, anddouble asterisks (**) indicate P < 0.01 vs. LPS group.

To further explore the mechanistic of alpinetin, the activation of TLR4 was evaluated by Western blot. Our results indicated that the expression of TLR4 was significantly increased by LPS, which were down-regulated by alpinetin in a dose-dependent manner (Fig. 9).

#### Effect of alpinetin on NLRP3 signaling pathway

**Figure 10:**
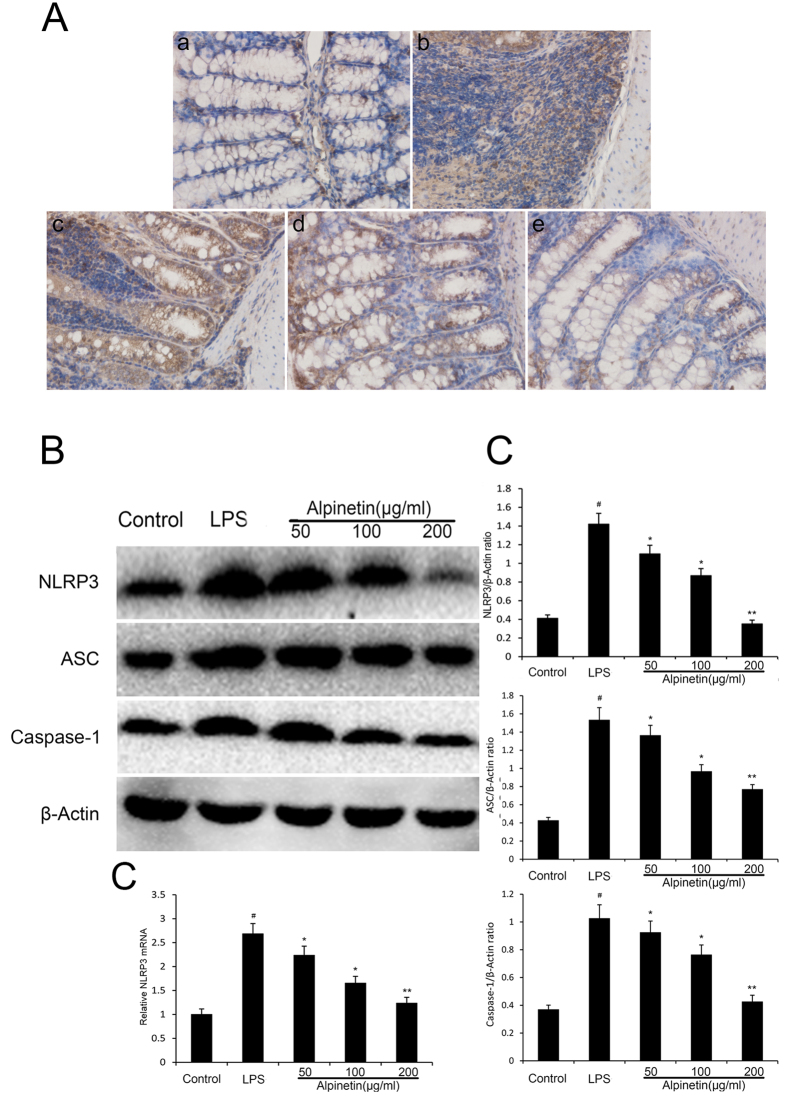
Alpinetin inhibited NLRP3 inflammasome activation. Colon tissue was collected to determine the NLRP3 positive signal by immunohistochemistry (**A**). Protein levels of NLRP3, ASC, caspase-1 in THP-1 cells were determined by Western blotting (**B**). The mRNA level of NLRP3 was determined by RT-PCR (**C**). The values presented are the mean ± S.E.M (n = 10 in each group).Number sign (#) indicates P < 0.01 vs. control group. Single asterisk (*) indicates P < 0.05, anddouble asterisks (**) indicate P < 0.01 vs. LPS group.

The NLRP3 inflammasome played an important role in many disorders^8^. Consequently, to elucidate whether its anti-inflammatory effect was related with NLRP3 inflammasome, we further explored the level of NLRP3 inflammasome in the colon and THP-1 cells. As shown in Fig. 10A, NLRP3 positive signals were obviously increased in the colon after DSS administration by immunohistochemical staining compared with the control group, while these changes were significantly attenuated through pretreatment with alpinetin. In addition, stimulation with LPS significantly increased the activation of NLRP3 in THP-1 cells at the genetic and protein levels. However, pretreatment with alpinetin significantly reduced the LPS-induced up-regulations of NLRP3 in THP-1 cells at the genetic and protein levels (Fig. 10B,C).

## Discussion

IBD is a chronic and relapsing inflammatory disorder of unknown etiology^9^, and affects millions of people worldwide. Alpinetin has been reported that it can inhibit inflammatory cytokines production and block the activation of NF-κB signaling^7^. In this research, we used the model of DSS-induced colitis mouse, the widely used model, to investigate the pathogenesis of IBD and the anti-inflammatory effect and mechanism of alpinetin via the inhibition of macrophage-mediated inflammation. The results of our study showed that alpinetin could reduce the inflammatory response of DSS-induced colitis in mice.

Our results showed that treatment with alpinetin suppressed DSS-induced colitis in mice through improving stool consistency and reducing body weight loss, bloody stool, and colon shortening. Histopathologic changes were an index of the response to the inflammation reactions^10^. We found that histopathologic findings confirmed that the administration of alpinetin protected DSS-induced colitis in mice from mucosal erosion, submucosal edema, inflammatory cell infiltration, and loss and disruption of crypts and villi. In addition, MPO is an enzyme that is directly proportional to the number of neutrophils in the tissue^11^. Decreased MPO activity illustrated the benefits of alpinetin treatment in the mouse model of DSS-induced colitis.

The pro-inflammatory cytokines of TNF-α and IL-1β, play important roles in the process of host defense and infection and inflammation pathological development, have been observed in many different types of inflammatory response, including colitis^12^,^13^. In particular, the binding of TNF-α and IL-1β to intestinal immune cells amplifies immune response by enhancing T cell proliferation, promoting leukocyte infiltration, and facilitating cell–cell signaling^14^. TNF-α, the earliest and primary endogenous produced mainly by macrophages, plays a central role in the inflammatory cascade^15^. IL-1β, a subtype of IL-1, is a pivotal inflammatory cytokine produced by both inflammatory cells and mucosal epithelial cells during colonic inflammation^16^. In the current study, alpinetin attenuated the release and mRNA levels of TNF-α and IL-1β from LPS primed PMA-differentiated THP-1 cells and inhibited DSS-induced TNF-α and IL-1β secretion from the colonic tissues of DSS-treated mice. IL-1β secretion was modulated through two signaling pathways. NF-κB signaling provides pro-IL-1β formation and NLRP3 inflammasome activation controls IL-1β cleavage from pro-IL-1β^17^,^18^. Several previous studies have demonstrated that the expressions of pro-inflammatory mediators are modulated by NF-κB pathway^19^,^20^. NF-κB, the most pro-inflammatory transcription factor of inflammatory factors, is involved in the regulation of inflammatory and immune responses and play an important role in the development of colitis^21^,^22^,^23^. Normally, NF-κB is sequestered in the cytoplasm by IκB proteins, which maintains the transcription factor in an inactive state^24^. In this study, alpinetin pre-treatment significantly inhibited the phosphorylation of IKKa/b, IjBa and p65 NF-κB activation in LPS-induced PMA-differentiated THP-1 cells.

Toll-Like Receptors (TLRs), evolutionarily conserved transmembrane receptors, were shown previously to play a central role in mucosal innate immune regulation^25^,^26^. TLR4, a member of the Toll-like family of proteins, localizes to both the cell membrane and the cytoplasm and is studied primarily in immune cells, which is a pattern recognition receptor for LPS^27^. Interaction of LPS with TLR4 and triggers signaling cascades initiates myeloid differentiation factor MyD88 activation of the downstream NF-κB signaling pathway and eventually results in inflammatory response^28^,^29^. In our study, TLR4 was upregulated in LPS-induced PMA-differentiated THP-1 cells; however, the upregulation of TLR4 was significantly reversed by alpinetin treatment.

Recent studies have suggested that NLRP3 governed the productions of pro-inflammatory cytokines, and is implicated in the pathogenesis of more common inflammatory diseases^30^,^31^. There is a close connection between NLRP3 inflammasome and NF-κB pathway^32^. Understanding the molecular mechanisms of activation has become a focus or research^33^. NLRP3 inflammasome, one of NLRs extensively studied in recent, has a basic structure consisting of NLRP3, the apoptosis-associated speck-like protein containing caspase-1 activator domain (ASC) adaptor recruited and activated procaspase-1, caspase-1^34^. The activation of caspase-1 is required to convert pro-IL-1β to its mature active form IL-1β, which was activated by LPS stimulation in macrophages via the activation of the NLRP3 inflammasome^35^. Upon activation, NLRP3 proteins combine to ASC adaptor and subsequently induce the translocation and activation of pro-caspase-1^36^. We observed that pretreated with alpinetin effectively reduced the levels of IL-1β in THP-1 cells, which was due to inhibit the expression of NLRP3, ASC and caspase-1.

In conclusion, the current study clearly demonstrates that alpinetin can effectively inhibit the expression of TNF-α and IL-1β in DSS-induced mouse colitis. The promising anti-inflammatory mechanism of alpinetin is associated with down-regulation of TLR4, NF-κB and NLRP3 inflammasome. Overall, these data collectively suggest that alpinetin may be a promising drug candidate for prophylaxis of colitis. To clarify the exact target of alpinetin as well as further molecular mechanism, more work should be done.

## Materials and Methods

### Animals

A total of 50 6-week-old female BALB/c mice were provided by the Center of Experimental Animals of Baiqiuen Medical College of Jilin University (Jilin, China). They were housed under a 12 h light/dark cycle at 24 ± 1 °C and 40–80% humidity and received food and water ad libitum for at least 7 days to adapt themselves to the environment before the experiments. All cages had been washed carefully and sterilized by autoclaving. All the animal experiments were performed in accordance with the experimental practices and standards approved by the Animal Welfare and Research Ethics Committee at Jilin University (approval ID 20111106–2), and all efforts were made to minimize suffering.

### Reagents

DSS was obtained from the Sigma Chemical Co. (L-2880, St.Louis, MO, USA). Alpinetin (purity 99.8%) was purchased from the National Institute for the Control of Pharmaceutical and Biological Products (Beijing, China). 3-(4,5-Dimethylthiazol-2-yl)-2,5-diphenyltetrazolium bromide (MTT), phorbol myristate acetate (PMA), dimethyl sulfoxide (DMSO) and LPS (E. coli 055:B5) were supplied by Sigma Chemical Company (St. Louis, MO, U.S.). RPMI 1640 medium, fetal bovine serum (FBS) and penicillin−streptomycin and sodium pyruvate solution were obtained from Hyclone (Logan, UT, USA). T-PER tissue protein extraction reagent (78510) was provided from Thermo. The MPO determination kit was purchased from the Jiancheng Bioengineering Institute of Nanjing (Nanjing, Jiangsu Province, China). All enzyme-linked immunosorbent assay (ELISA) kit was purchased from Biolegend (USA). All the monoclonal antibodies which were used in Western blot were obtained from Cell Signaling Technology Inc (Beverly, MA, USA). All other chemicals were of reagent grade.

### *In vivo* study

#### Experiment Model and Experimental Protocol

Experimental colitis was induced by giving mice drinking water containing 5% (w/v) DSS for 7 days. Mice were randomly divided into 4 groups (n = 10/group), including blank control group, DSS group, DSS + alpinetin groups (25, 50 and 100 mg/kg). Alpinetin (25, 50 and 100 mg/kg) were administered once a day by intraperitoneal injection 3 days before DSS treatment, and the blank control group and DSS group were administered with an equal volume of phosphate buffered saline (PBS). At day 7 following induction with DSS, the mice were killed by CO_2_ inhalation and then the entire colon were collected and stored at −80 °C until analysis.

#### Evaluation of Disease activity indices (DAI)

The disease activity index (DAI) was calculated by assigning well-established and validated scores as previously described^37^. The mice was macroscopically assessed based on body weight loss, stool consistency, and gross bleeding. Briefly, a) body weight loss: 0 = none; 1 = 1–5%; 2 = 5–10%; 3 = 10–15%; 4 = over 15%; b) stool consistency: 0 = normal; 2 = loose stools; 4 = diarrhea; c) gross bleeding: 0 = normal; 2 = hemoccult; 4 = gross bleeding^38^. Colonic shortening was determined by measuring the length between the ileo–cecal junction and the proximal rectum.

#### Histological examination of colon

For histological evaluation, the entire colon were collected and fixed in 10% buffered formalin for 24 h at room temperature, imbedded in paraffin and sliced. Samples were sectioned into 5 μm slices and subjected to staining with hematoxylin and eosin (H.E). Finally, the section was examined under a light microscopy for evaluating the histopathologic changes of the colon tissues. Colonic damage was scored as described previously^39^.

#### Immunohistochemical Staining

For immunohistochemical staining, slices of colon were snapfrozen in optimal cutting temperature (OCT) solution and 5 μm sections of tissues were utilized. The sections were deparaffinized with xylene and rehydrated with ethanol. After inhibiting endogenous peroxidase using 3% H_2_O_2_ in methanol, the sections were rinsed with PBS, and then incubated with rabbit polyclonal antibody against NF-κB and NLRP3 (Santa Cruz Biotechnology; dilution 1:100) overnight at 4 °C and the secondary antibody at room temperature for 30 min. Reaction products were visualized following incubation with diaminobenzidine as chromogen and counterstaining with haematoxylin (Sigma Chemical Co.). Negative controls were generated by omitting the primary antibodies.

#### MPO assay

MPO activity is a mark of neutrophils in the tissue, which reflects the number and distribution of neutrophils in the tissus. The colon tissues were weighed and homogenized with PBS (1:9, w/v). The supernatants were collected. The activity of MPO was measured according to the manufacturer’s instructions.

#### Analysis of cytokine levels

The colon tissues were weighed and homogenized with PBS (1:9, w/v). The supernatants were collected. The expression of TNF-α and IL-1β were detected by ELISA according to the manufacturer’s instructions.

### *In vitro* study

#### Cell culture and treatment

Human THP-1 cells were purchased from Shanghai Institute of Cell Biology (Shanghai, China) and cultured in RPMI 1640 media with 10% (v/v) FBS, 100 μg/mL streptomycin, 100 U/ml penicillin. Culture fluid was replaced every 2 ~ 3 days, and the cultures were maintained at 37 °C, 95% air/5% CO_2_ in a fully humidified incubator. To mimicking the activation of NLRP3 inflammasome *in vitro*, THP-1 cells was induced by 0.5 μM PMA for 3 h, as described previously^40^. THP-1 cells were primed with LPS (5 μg/ml; 4 h) in serum-free medium. Alpinetin (100, 200 μg/ml) were added 1 h before LPS.

#### Cell viability assay

The cytotoxic effects of alpinetin to THP-1 cells were assessed by MTT assay. Briefly, the cells were seeded at a density of 2 × 10^5^ cells/ml or 4 × 10^5^ cells/ml in 96-well plates in a 37 °C, 5% CO_2_ incubator for 1 h, then the cells were treated with 50 μl of aipinetin at different concentrations (0 ~ 200 μg/ml) for 24 h. Then, 20 μl of MTT (5 mg/ml) was added for 3 h. The supernatant was discarded, and 150 μl per well of DMSO was added. The optical density was measured at 570 nm on a microplate reader (TECAN, Austria).

#### Cytokine assay

THP-1 cells were seeded in a 6-well-plate, and the cells are treated as indicated. Cytokines, TNF-α and IL-1β in the cell supernatants, were measured using commercially available ELISA kit, according to the manufacturer’s instructions.

#### Real-time PCR

THP-1 cells were plated at a density of 1 × 10^6^ cells/ml in 6-well plates at 37 °C with 5% CO_2_ for 24 h, and the cells are treated as indicated. Total RNA in THP-1 cells was extracted using TRIzol (Invitrogen, Carlsbad, CA, USA) and treated with DNase I (MBI Fermentas, Lithuania) to remove genomic DNA contamination. Real-time PCR (RT-PCR) was performed according to the manufacturer’s instructions of the Revert Aid First Strand cDNA Synthesis Kit (MBI Fermentas, Lithuania) as described in our previous research 41. The primers were acquired from Sangon Biotech Co. Ltd (Shanghai, China), which sets used: TNF-α sense, 5′-ACGGGCTTTACCTCATCTACTC-3′, TNF-α anti-sense, 5′-GCTCTTGATGGCAGACAGG-3′; IL-1β sense, 5′-AGGTGGTGTCGGTCATCGT-3′, IL-1β anti-sense, 5′-GCTCTCTGTCCTGGAGTTTGC-3′.

#### Western blot analysis

Total proteins were extracted from the cells using the M-PER Mammalian Protein Extraction Reagent. The protein concentration was determined by the BCA method and separated by 10% SDS polyacrylamide gels and transferred to a polyvinylidene difluoride membrane. In the next step, the membranes were blocked with 5% skim milk in Tris–Tween-buffered saline (TBST) for 2 h at room temperature, and incubated overnight at 4 °C with primary antibodies (1:1000 dilutions in TBST). Subsequently, the membranes were incubated with peroxidase-conjugated secondary antibody (1:50000 dilutions in TBST) and then visualized with the ECLPlus Western Blotting Detection System (GE Healthcare, Chalfont St Giles, UK).

#### Statistical Analysis

All of the data in this study were presented as means ± S.E.M. Differences between the mean values of normally distributed data were assessed by one-way ANOVA (Dunnett’s t test) and the two-tailed Student’sttest. Values of P < 0.05 were considered to be statistically significant.

## Additional Information

**How to cite this article**: He, X. *et al.* Alpinetin attenuates inflammatory responses by suppressing TLR4 and NLRP3 signaling pathways in DSS-induced acute colitis. *Sci. Rep.* **6**, 28370; doi: 10.1038/srep28370 (2016).

## Figures and Tables

**Figure 1:**
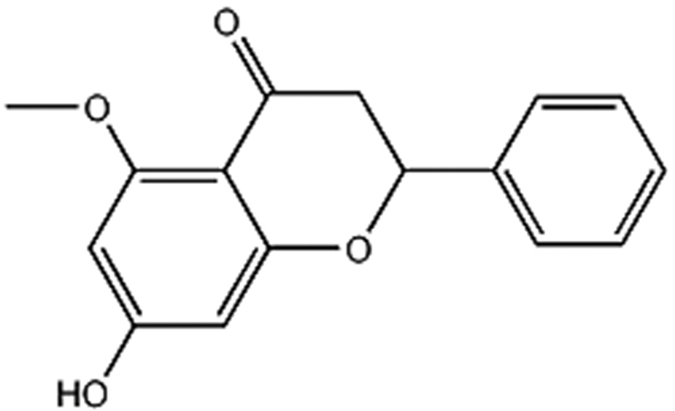
The chemical structure of alpinetin.
